# Residues 318 and 323 in capsid protein are involved in immune circumvention of the atypical epizootic infection of infectious bursal disease virus

**DOI:** 10.3389/fmicb.2022.909252

**Published:** 2022-07-29

**Authors:** Linjin Fan, Yulong Wang, Nan Jiang, Yulong Gao, Xinxin Niu, Wenying Zhang, Mengmeng Huang, Keyan Bao, Aijing Liu, Suyan Wang, Li Gao, Kai Li, Hongyu Cui, Qing Pan, Changjun Liu, Yanping Zhang, Xiaomei Wang, Xiaole Qi

**Affiliations:** ^1^Avian Immunosuppressive Diseases Division, State Key Laboratory of Veterinary Biotechnology, Harbin Veterinary Research Institute, The Chinese Academy of Agricultural Sciences, Harbin, China; ^2^OIE Reference Laboratory for Infectious Bursal Disease, Harbin Veterinary Research Institute, The Chinese Academy of Agricultural Sciences, Harbin, China; ^3^National Laboratory of Biomacromolecules, CAS Center for Excellence in Biomacromolecules, Institute of Biophysics, Chinese Academy of Sciences, Beijing, China; ^4^Jiangsu Co-innovation Centre for Prevention and Control of Important Animal Infectious Disease and Zoonoses, Yangzhou University, Yangzhou, China

**Keywords:** atypical infectious bursal disease, epizootic varIBDV, antigenicity difference, immune circumvention, VP2

## Abstract

Recently, atypical infectious bursal disease (IBD) caused by a novel variant infectious bursal disease virus (varIBDV) suddenly appeared in immunized chicken flocks in East Asia and led to serious economic losses. The epizootic varIBDV can partly circumvent the immune protection of the existing vaccines against the persistently circulating very virulent IBDV (vvIBDV), but its mechanism is still unknown. This study proved that the neutralizing titer of vvIBDV antiserum to the epizootic varIBDV reduced by 7.0 log_2_, and the neutralizing titer of the epizootic varIBDV antiserum to vvIBDV reduced by 3.2 log_2_. In addition, one monoclonal antibody (MAb) 2-5C-6F had good neutralizing activity against vvIBDV but could not well recognize the epizootic varIBDV. The epitope of the MAb 2-5C-6F was identified, and two mutations of G318D and D323Q of capsid protein VP2 occurred in the epizootic varIBDV compared to vvIBDV. Subsequently, the indirect immunofluorescence assay based on serial mutants of VP2 protein verified that residue mutations 318 and 323 influenced the recognition of the epizootic varIBDV and vvIBDV by the MAb 2-5C-6F, which was further confirmed by the serial rescued mutated virus. The following cross-neutralizing assay directed by MAb showed residue mutations 318 and 323 also affected the neutralization of the virus. Further data also showed that the mutations of residues 318 and 323 of VP2 significantly affected the neutralization of the IBDV by antiserum, which might be deeply involved in the immune circumvention of the epizootic varIBDV in the vaccinated flock. This study is significant for the comprehensive prevention and control of the emerging varIBDV.

## Introduction

Infectious bursal disease (IBD), an acute, highly contagious and immunosuppressive disease in chickens, has been threatening poultry farming worldwide (Müller et al., 2003; Jackwood, 2017). IBD is caused by the infectious bursal disease virus (IBDV), an RNA virus that belongs to the genus *Avibirnavirus* of the family *Birnaviridae*. The IBDV has a non-enveloped capsid structure containing a double-stranded RNA genome with two segments, A and B (Brown and Skinner, 1996; Müller et al., 2003). Segment A encodes four viral proteins that include two structural proteins VP2 and VP3, a viral protease VP4, and a nonstructural protein VP5 (Raja et al., 2016). The capsid protein VP2 is located on the surface of the virus, and its outermost surface is composed of four loop regions, namely, P_BC_ (aa 204-236), P_DE_ (aa 240-265), P_FG_ (aa 270-293), and P_HI_ (aa 305-337) (Coulibaly et al., 2005). In addition to VP2 being the icosahedral capsid protein, it is also the major protective immunogen of the IBDV and the primary determinant of viral virulence and antigenic variation (Brandt et al., 2001; Qi et al., 2016). Segment B is approximately 2.8 kb and contains only one open reading frame (ORF), which encodes viral protein VP1. As an RNA-dependent RNA polymerase (RdRp), VP1 plays important role in viral replication and genetic evolution (Escaffre et al., 2013; Yu et al., 2013; Gao et al., 2014).

The IBDV has two serotypes. Serotype I includes virus strains that are pathogenic to chickens, whereas serotype II viruses, isolated from turkeys, are apathogenic to chickens. Since the identification of the classic strain during the first outbreak of IBD in 1957 (Cosgrove, 1962), a variant IBDV (varIBDV) in North America (Jackwood and Saif, 1987) and a very virulent IBDV (vvIBDV) in Europe (Chettle et al., 1989) have successively emerged. The varIBDV of the genotype A2aB1/A2bB1/A2cB1 (the early varIBDV) has always been the circulating strain in North America (Jackwood and Sommer-Wagner, 2005; Kurukulsuriya et al., 2016; Wang Y. et al., 2021). However, the vvIBDV, characterized by acute death, quickly swept the world and became one of the important threats to the poultry industry in the past 30 years (van den Berg, 2000; Müller et al., 2003; Jackwood, 2017; de Wit et al., 2018; Wang Y. et al., 2021). Since 2017, atypical IBD infection, widespread in immunized chicken flocks in East Asia (Fan et al., 2019; Xu G. et al., 2019; Li et al., 2020; Aliyu et al., 2021; Myint et al., 2021; Thai et al., 2021; Wang Y. et al., 2021), has been causing serious economic losses. Our laboratory identified its pathogen as a novel variant IBDV of genotype A2dB1 (hereinafter referred to as the epizootic varIBDV) for the first time, which is genetically different from the early varIBDV in North America (Fan et al., 2019; Wang Y. et al., 2021). The newly epizootic varIBDV and the persistently circulating vvIBDV are the two dominant strains, especially in China (Jiang et al., 2021).

The epizootic varIBDV can partly break through the immune protection of the existing vaccine against the vvIBDV, which is one of the important factors for the virus to become widespread in immunized chickens (Fan et al., 2020a; Li et al., 2020). It was speculated that the epizootic varIBDV might have antigenicity differences compared with vvIBDV. This study proved this speculation and revealed that residues 318 and 323 of the capsid protein VP2 were deeply involved in the antigenicity difference between the epizootic varIBDV and vvIBDV, which would provide important insights into viral evolution and comprehensive prevention of the IBDV.

## Materials and methods

### Cells, viruses, antibodies and plasmids

DF-1 cells were cultured in Dulbecco's modified Eagle medium (DMEM) (Invitrogen, USA) supplemented with 10% fetal bovine serum (FBS) at 37°C in a humidified incubator with 5% CO_2_. DT40 cells were cultured in Roswell Park Memorial Institute (RPMI) 1,640 Medium (Invitrogen, USA) with 10% FBS, 1% glutamine, 2% chicken serum, and 0.1% β-mercaptoethanol at 37°C in a humidified incubator. The epizootic varIBDV representative strain SHG19 (Fan et al., 2020b,c) was previously isolated and identified by the Avian Immunosuppressive Disease Laboratory, Harbin Veterinary Research Institute (HVRI), Chinese Academy of Agricultural Sciences (CAAS) (hereinafter referred to as “our lab”). The Chinese vvIBDV representative strains HLJ0504 (Qi et al., 2011) and Gx (Wang et al., 2004) also were previously identified by our lab. The monoclonal antibodies (MAbs) against IBDV VP2 (2-5C-6F and 7D4) were developed by our lab. The MAb 2-5C-6F can neutralize the vvIBDV, but not 7D4. The eukaryotic expression vector pCAGGS (Niwa et al., 1991; van den Berg et al., 1991) was kindly supplied by Dr. J. Miyazaki, University of Tokyo, Tokyo, Japan. The infectious clones pCAmGtAHRT and pCAmGtBHRT, each containing segments A and B of attenuated IBDV Gt flanked by ribozyme sequences, were previously constructed (Qi et al., 2007).

### Chickens

Specific pathogen-free (SPF) chickens were purchased from the Experimental Animal Center of the HVRI of the CAAS (Harbin, China) and were housed in negative pressure-filtered air isolators. All the animal experiments were approved by the HVRI of the CAAS and were performed according to the animal ethics guidelines and approved protocols.

### Indirect immunofluorescence assay

Indirect immunofluorescence assay (IFA) directed by MAbs 7D4 and 2-5C-6F against IBDV VP2 was performed to determine the antigenicity difference between the epizootic varIBDV and vvIBDV. For the IFA, DT40 cells or DF1 cells, cultured in a 24-well tissue culture plate, were separately infected with IBDVs (200 TCID_50_/well) (50% tissue culture infective dose) or transfected with recombinant plasmids (1 μg/well). Cells treated with PBS were used as a negative control. At 24 h post-infection or 48 h post-transfection, the cells in different wells were fixed and then incubated with MAbs (2-5C-6F or 7D4) for 1 h, followed by staining with a fluorescein-labeled goat anti-mouse antibody (1:200 dilution) (Sigma, USA) for another 1 h. The cells were examined by fluorescence microscopy after washing five times with PBS. The quantitative analysis of fluorescence was performed as described previously (Jensen, 2013).

### Serum cross-neutralization assay

The serum cross-neutralization assay was performed using the antiserum against each virus strain to further evaluate the antigenicity difference between the epizootic varIBDV and vvIBDV and explore its key residues. First, the TCID_50_ of IBDV strains was determined in DT40 cells by IFA using an IBDV-specific MAb, then IBDV strain was diluted with 10-fold serial dilutions, and each dilution had 8-well repeats. The TCID_50_ detection was performed three times. Then 200 TCID_50_ of viruses were incubated for 1 h at 37°C with equal volumes of antiserum at 2-fold serial dilutions. The virus–serum mixture (100 μl) was cultured with DT40 cells at 37°C in a humidified incubator. After 24 h, the infection-positive wells were detected by IFA, and the homologous or heterologous neutralization titers were determined. Each neutralization assay was performed at least three times.

### Identification of the antigen epitopes

A series of overlapping VP2 peptides of the vvIBDV Gx strain were cloned into pGEX-6P-1 and expressed in *E. coli* BL21 (DE3) as fusion proteins with a GST tag to investigate the epitopes of the MAbs 2-5C-6F or 7D4. For the first round, three overlapping peptides spanning the VP2 amino acid (aa) sequences, aa 1-167 (VP2A), aa 141-305 (VP2B), aa 261-441 (VP2C), were expressed in *E. coli*. The VP2X was further divided into three peptides for the second round of screening. For the last round, the peptide amino acids were truncated one by one from both ends until the minimal epitope was identified. The schematic diagrams of all peptides are listed in **Figure 2A**, and the primers are shown in Supplementary Table 1. The reactivity of the MAbs was detected by Western blot (WB), and the IRDye 800CW goat anti-mouse antibody (LI-COR) was used as the secondary antibody.

### Epitope sequence alignment

The amino acid sequence of the VP2, containing the identified epitopes, was compared with that in other subtypes of serotype I of IBDV, including classic, very virulent, attenuated, and variant strains, using MegAlign software (DNASTAR, Madison, WI, USA). The three-dimensional (3-D) structure of the VP2 protein of the IBDV SHG19 strain was predicted by using the I-TASSER algorithm (Yang et al., 2015). The predicted structure of SHG19 VP2 was compared with that of VP2 of the vvIBDV Gx strain (Bao et al., 2021) using PyMOL software (http://pymol.org/).

### Construction of the mutated VP2

The major protective antigen gene VP2 of SHG19 and Gx strains were amplified with primers VP2F/VP2R (Table 1) and then cloned into the plasmid pCAGGS, and the recombinant plasmid was named pCASHG19VP2 and pCAGxVP2. Using PCR for site-directed mutagenesis as described previously (Qi et al., 2007), direct mutations were introduced into the VP2 gene of the SHG19 and Gx strains. Based on the plasmid pCASHG19VP2, primer pairs 19-318F/318R and 19-323F/323R (Table 1) were used to introduce direct mutations A953G, A969C, and A953G/A969C (which resulted in amino acid mutations of D318G, E323D, and D318G/E323D in VP2) into the VP2 gene of the SHG19 strain, and the mutated plasmids were named pCASHG19VP2-D318G, pCASHG19VP2-E323D, and pCASHG19VP2-D318G/E323D. Based on the plasmid pCAGxVP2, primer pairs Gx-318F/318R and Gx-323F/323R (Table 1) were used to introduce mutations using the same approach, and the mutated plasmids were named pCAGxVP2-G318D, pCASHG19VP2-D323E, and pCASHG19VP2-G318D/D323E.

**Table 1:** Primers.

**Primer**	**Sequence**	**Orientation**	**Position (nt)**
A1	**AAAGAATTCGATCTC**ATCGATTGTTAAGCGTCTGAT	Sense	A: −58 to −44
A2	AGACCGATCGTATCCGACTATAGGAATTC	Antisense	A: 15 to −14
A3	GGAATTCCTATAGTCGGATACGATCGGTCT	Sense	A: −15 to 15
A4	ATGCCATGCCGACCCGGGGACCCGCGAACG	Antisense	A: +15 to 3245
A5	CGTTCGCGGGTCCCCGGGTCGGCATGGCAT	Sense	A: 3245 to +15
A6	**GCTCGAGCATGCCCG**GGTACCCGCCCTCCCTTAGC	Antisense	A: +88 to +75
B1	**TTTGGCAAAGAATTC**GAGCTCTGTTAAGCGTCTGAT	Sense	B: −58 to −44
B2	CAGACCCATCGTATCCGACTATAGGAATTCC	Antisense	B: 16 to −15
B3	GGAATTCCTATAGTCGGATACGATGGGTCTG	Sense	B: −15 to 16
B4	GATGCCATGCCGACCCTTGGGGGCCCCCGC	Antisense	B: +18 to 2,816
B5	GCGGGGGCCCCCAAGGGTCGGCATGGCATC	Sense	B: 2,816 to +18
B6	**ATCTGCTAGCTCGA**GCATGCCGCCCTCCCTTAGCCAT	Antisense	B: +88 to +72
19–318F	GTGACCTCCAAAAGTG***G***TGGCCAGGCAGGGG	Sense	A:1,067 to 1,097
19–318R	GTTCCCCTGCCTGGCCA***C***CACTTTTGGAGGTCACTA	Antisense	A: 1,100 to 1,065
19–323F	GATGGCCAGGCAGGGGA***C***CAGATGTCGTGGTC	Sense	A: 1,082 to 1,113
19–323R	GCCGACCACGACATCTG***G***TCCCCTGCCTGGCCATCAC	Antisense	A: 1,116 to 1,080
Gx−318F	TAACCTCCAAAAGTG***A***TGGTCAGGCGG	Sense	A: 1,068 to 1,094
Gx−318R	TCCCCCGCCTGACCA***T***CACTTTTGGAGGTTAC	Antisense	A: 1,098 to 1,067
Gx−323F	TCAGGCGGGGGA***A***CAGATGTCATGG	Sense	A: 1,087 to 1,111
Gx−323R	TGACCATGACATCTG***T***TCCCCCGCCTGACC	Antisense	A: 1,114 to 1,085
VP2F	**AAAGAATTCGATCTC**GGATCCATGACAAACCTGCAAGATC	Sense	A: 131 to 149
VP2R	**TAGCTCGAGCATGCCCG**AAGCTT TGCTCCTGCAATCTTC	Antisense	A: 1,453 to 1,438

Primers were designed according to the sequence of IBDV strain Gx (GenBank accession no. AY444873) and SHG19 (GenBank accession no. MN393076, MN393077). The ribozyme sequences are surrounded by boxes, the introduced restriction sites are underlined, the homology arms sequences are boldfaced, and the mutated nucleotides are shown in italic and bold. Orientation and position of the virus-specific sequences of the primers are shown. The “+” or “–” symbols in front of the positions of nucleotides indicate the upstream or downstream of the genome, respectively. “A” and “B” in the last column represent segments A and B of IBDV genome.

### Rescue of the mutated IBDV

A fusion PCR was performed as described previously (Qi et al., 2009) to construct infectious clones of the SHG19 strain. First, with pCAmGtAHRT as a template, two PCR fragments, F1 and F3, were amplified with two pairs of primers (A1/A2 and A5/A6 in Table 1), and the lengths of PCR products were 94 bp and 120 bp, respectively. Second, with the SHG19 strain as a template, the PCR fragment F2 of 3290 bp was amplified with primers A3/A4 (Table 1). Finally, a fragment SHG19AHRT was fused by three fragments F1, F2, and F3. The purified PCR product SHG19AHRT was digested with *Cla* I/*Kpn* I and ligated into pCAGGS to obtain the infectious clone pCASHG19AHRT of segment A. Based on the plasmid SHG19AHRT, primer pairs 19-318F/318R and 19-323F/323R (Table 1) were used to introduce direct mutations, and the mutated plasmids were named pCASHG19A-A953GHRT, pCASHG19A-A969CHRT, and pCASHG19A-A953G/A969CHRT. Simultaneously, with pCAmGtBHRT as a template, the fragment SHG19BHRT was amplified by three pairs of primers (B1/B2, B3/B4, and B5/B6), as given in Table 1. The purified PCR product SHG19BHRT was digested with *Sac* I/*Sph* I and ligated into pCAGGS to obtain the infectious clone pCASHG19BHRT of segment B. The schematic diagrams of infectious clones are shown in **Figure 4A**.

Mutated IBDVs were rescued by using an RNA polymerase II-driven reverse genetic system (Qi et al., 2007). The purified infectious clone plasmids of segment A and segment B (2 μL of each plasmid at 1 μg/μL) were co-transfected into DF-1 cells using a TransIT-X2 Dynamic Delivery System (Mirusbio, Madison, Wisconsin, USA). At 72 h post-transfection, after freezing and thawing three times, the cell suspension was injected into bursae of 14-day-old SPF chickens. At 7 d post-injection, the bursal tissues were collected, and then the rescued viruses were detected. To characterize the rescued viruses, a fragment of 930 bp was amplified by RT-PCR using primer pairs 2U/2L (bp 628-1557 of segment A) and then sequenced (Fan et al., 2019). The full-length genomes of the rescued viruses were further amplified and sequenced to confirm the accuracy as designed. The rescued viruses were also identified by IFA directed by MAbs 7D4 in DT40 cells. The replication of the rescued viruses in DT40 cells was detected at 24, 36, 48, and 60 h post-infection by RT-qPCR, as previously described (Wang S. et al., 2021). The correctly identified rescued viruses were named SHG19, SHG19-318, SHG19-323, and SHG19-318/323, respectively.

### Statistical analysis

The significance of the variability between different groups was determined by two-way analysis of variance (ANOVA), using GraphPad Prism software (version 8.0). Significant treatment means were separated using Tukey's honestly significant difference (Tukey's HSD) at *P* = 0.05.

## Results

### The antigenicity of the epizootic varIBDV is different from vvIBDV

**Figure 1:**
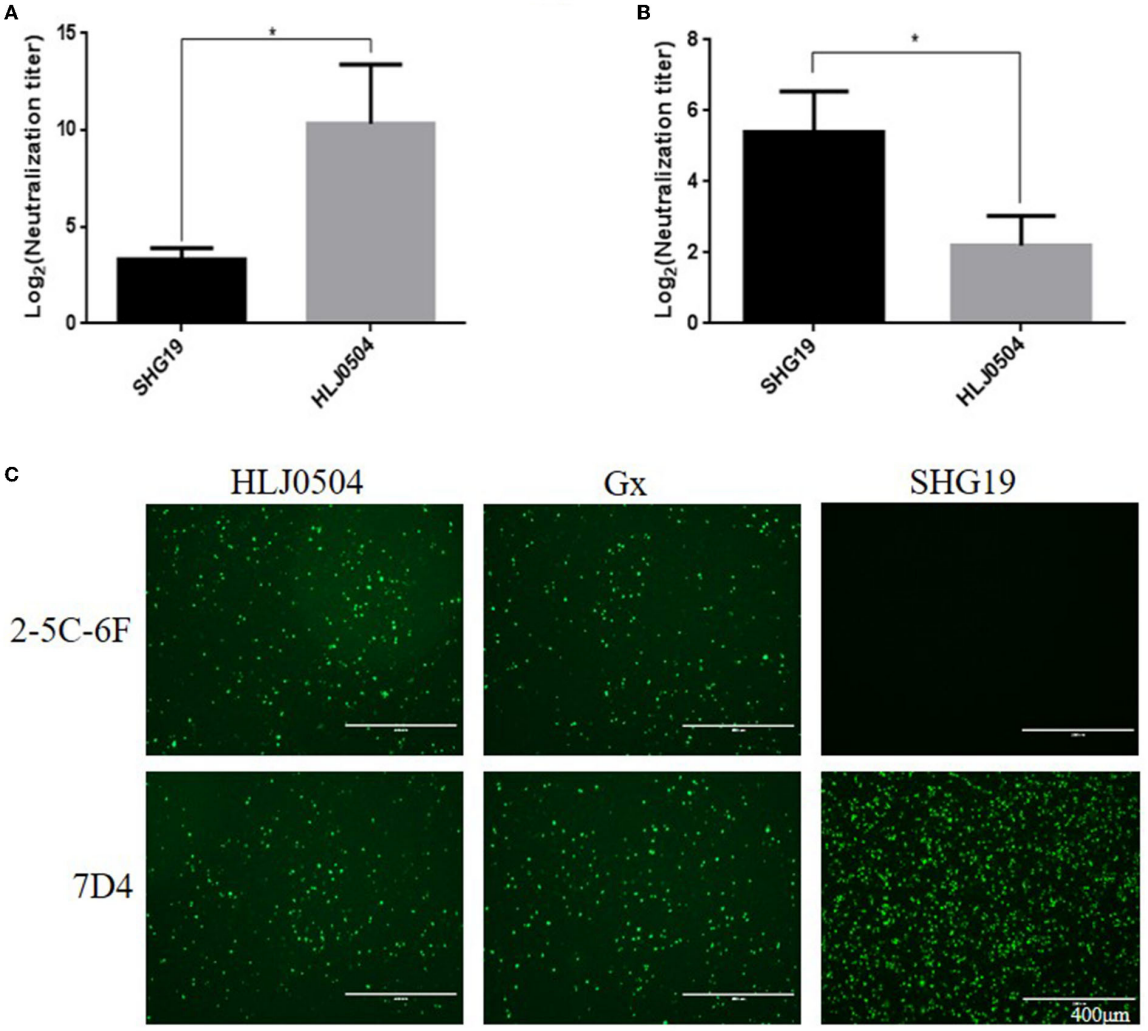
Detection of antigenicity difference between the epizootic varIBDV (SHG19 strain) and vvIBDV (HLJ0504 strain and Gx strain). **(A)** Cross-neutralizing assay of HLJ0504 antiserum for SHG19 and HLJ0504 on DT40 cells directed. **(B)** Cross-neutralizing assay of SHG19 antiserum for SHG19 and HLJ0504 on DT40 cells directed. **(C)** IFA on DT40 cells directed by MAbs 2-5C-6F and 7D4. The mean titers and standard deviations (error bars) from three **(A)** or five **(B)** independent samples are shown. * represents *p* < 0.05.

To determine the antigenicity difference between the epizootic varIBDV and vvIBDV, the cross-neutralizing assay for the varIBDV representative strain SHG19 and the vvIBDV representative strain HLJ0504 was performed. The results indicated that HLJ0504 antiserum had a high homologous neutralization titer against HLJ0504 at 10.33 ± 3.06 log_2_, but its heterologous neutralization titer (3.33 ± 0.58 log_2_) for SHG19 reduced by 7.0 log_2_ (Figure 1A). Similarly, SHG19 antiserum had a homologous neutralization titer against SHG19 at 5.40 ± 1.14 log_2_, but its heterologous neutralization titer (2.20 ± 0.84 log_2_) for HLJ0504 reduced by 3.2 log_2_ (Figure 1B). In addition, with IFA in DT40 cells, the MAbs against IBDV VP2 (2-5C-6F and 7D4) were used to further detect the antigenicity difference between the epizootic varIBDV (SHG19) and vvIBDV (HLJ0504 and Gx). The results showed that all the epizootic varIBDVs (SHG19) and vvIBDVs (HLJ0504 and Gx) could be recognized by MAb 7D4, while the MAb 2-5C-6F could only recognize vvIBDV (HLJ0504 and Gx), but not the epizootic varIBDV (SHG19) (Figure 1C). These results confirmed that the antigenicity of the epizootic varIBDV was different from that of vvIBDV.

### The antigen epitopes of IBDV were identified by neutralizing MAb

**Figure 2:**
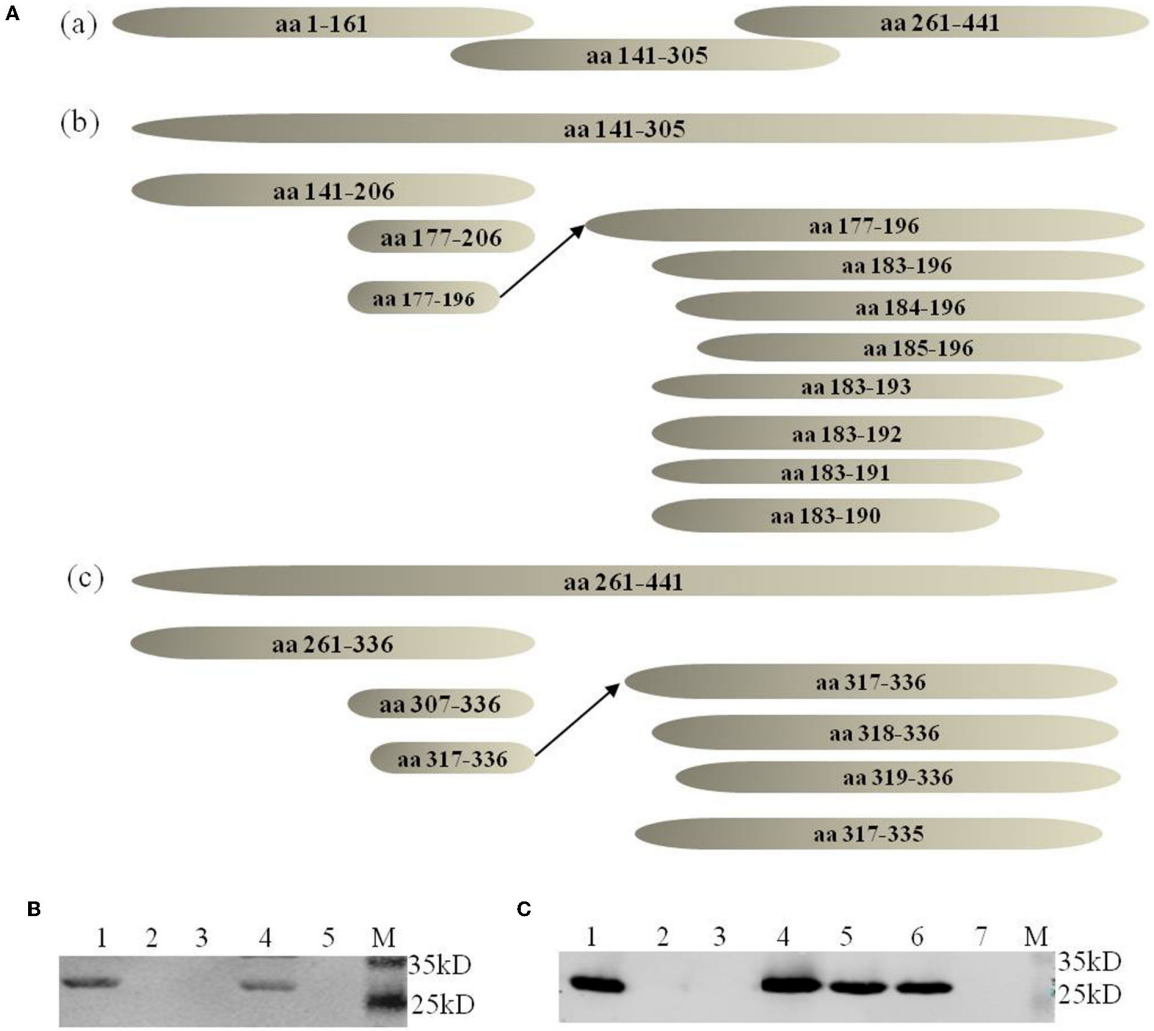
Identification of antigen epitopes with MAbs 7D4 and 2-5C-6F. **(A)** Schematic diagrams of all peptides (not drawn to scale) used in the peptide-scanning method. (a) Three overlapping peptides spanning the VP2 aa sequences. (b) Identification of the antigen epitope with MAb 7D4. (c) Identification of the antigen epitope with the MAb 2-5C-6F. **(B)** Recognition of the minimal epitope with 2-5C-6F by Western blotting. Lane 1 corresponds to the epitope of aa 317-336; 2, aa 318-336; 3, aa 319-336, 4 corresponds to aa 317-336; 5, aa 317-335; M, marker. **(C)** Recognition of the minimal epitope with MAb 7D4 by Western blotting. 1, aa 183-196; 2, aa 184-196; 3, aa 185-196; 4, aa 183-193, 5, aa 183-192, 6, aa 183-191; 7, aa 183-190; M, marker.

**Figure 3:**
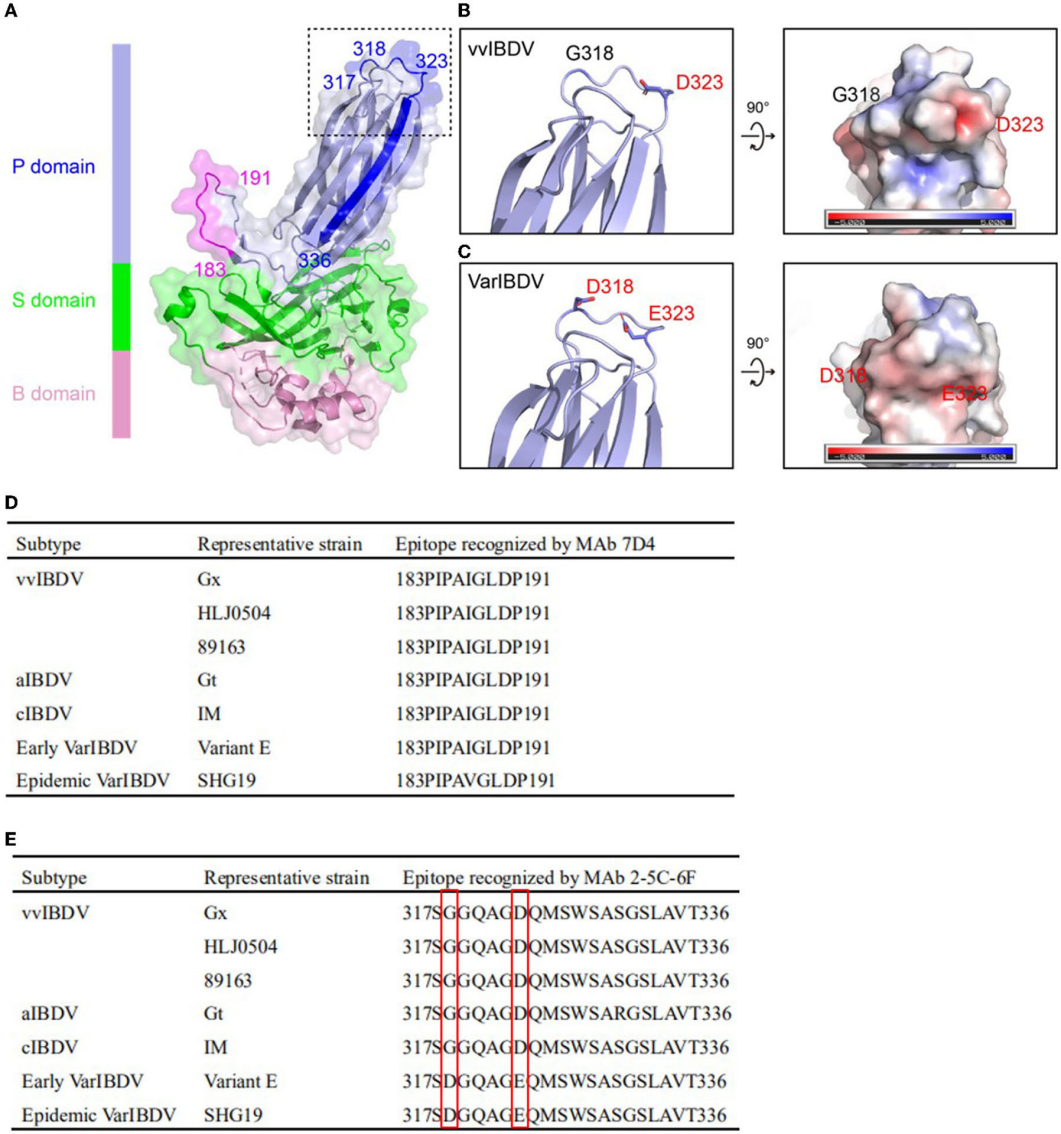
Location and sequence alignment of MAb antigenic epitopes. **(A)** Structure of IBDV VP2. P, S, and B domains were marked with different colors. The antigen epitopes of MAbs 7D4 (blue or magenta) and 2-5C-6F (blue or magenta) and the key residues are highlighted, respectively. The antigen epitopes of MAbs 7D4 and 2-5C-6F and key amino acid residues are highlighted. **(B)** Left: Zoom-in view of dashed box in **(A)** showing the key residues 318 and 323 of vvIBDV VP2. Right: 90° rotated view of the left panel showing the electrostatic surface representation of the vvIBDV. The side chains of residues 318 and 323 are displayed as a stick. **(C)** Left: Zoom-in view of dashed box in **(A)** showing the key residues 318 and 323 of varIBDV VP2 predicted by using the I-TASSER algorithm. Right: 90° rotated view of the left panel showing the electrostatic surface representation of varIBDV. The side chains of residues 318 and 323 are displayed as stick. **(D)** Amino acid sequence of the antigen epitope recognized by MAb 7D4 among different subtype strains of IBDV. **(E)** Amino acid sequence of the antigen epitope recognized by the MAb 2-5C-6F among different subtype strains of IBDV. The amino acid residues 318 and 323 of the antigen epitope recognized by the MAb 2-5C-6F are highlighted.

The neutralizing MAb 2-5C-6F of vvIBDV could not well recognize the epizootic varIBDV SHG19 by IFA. To explore the molecular basis, the antigen epitope recognized by MAb was identified step by step by using the peptide-scanning method based on a series of overlapping VP2 (Figure 2A). First, it was identified that the MAb 2-5C-6F and MAb 7D4 reacted with an epitope of aa 261-441 and an epitope of aa 141-305 (data not shown). Then the epitope of aa 261-441 was gradually truncated, and the MAb 2-5C-6F targeting antigen epitope was identified as aa 317-336 of VP2 (Figure 2B), which is located in the P_HI_ loop of VP2 (Figure 3A). Simultaneously, the epitope of aa 141-305 was gradually truncated, and the antigen epitope targeted by MAb 7D4 was determined as aa 183-191 of VP2 (Figure 2C), which is located outside of the projection (P) domain of VP2 (Figure 3A).

Sequence analysis showed that the epitope recognized by 7D4, 183PIPAIGLDPKM191, was conserved for all IBDV subtypes of serotype 1 (Figure 3D). However, for the epitope recognized by 2-5C-6F (317SGGQAGDQMSWSASGSLAVT336), both the epizootic varIBDV and the early varIBDV were different from other IBDV subtypes. The varIBDV showed 318D and 323E, but other IBDV subtypes including vvIBDV showed 318G and 323D (Figure 3E). The alignment results indicated that residues 318D and 323E are conserved in different varIBDV strains isolated from broiler, layer, and local breed chickens (Supplementary Figure 1). According to the predicted VP2 structure, the mutations G318D and D323E might alter the structure of the extreme outermost region of the VP2 P domain and the associated electrostatic potential (Figures 3B,C). Especially the G318D mutation presents a negatively charged surface at the region of SHG19 (Figure 3C).

### Residue mutations of 318 and 323 of VP2 influence the recognition of IBDV by MAb

**Figure 4:**
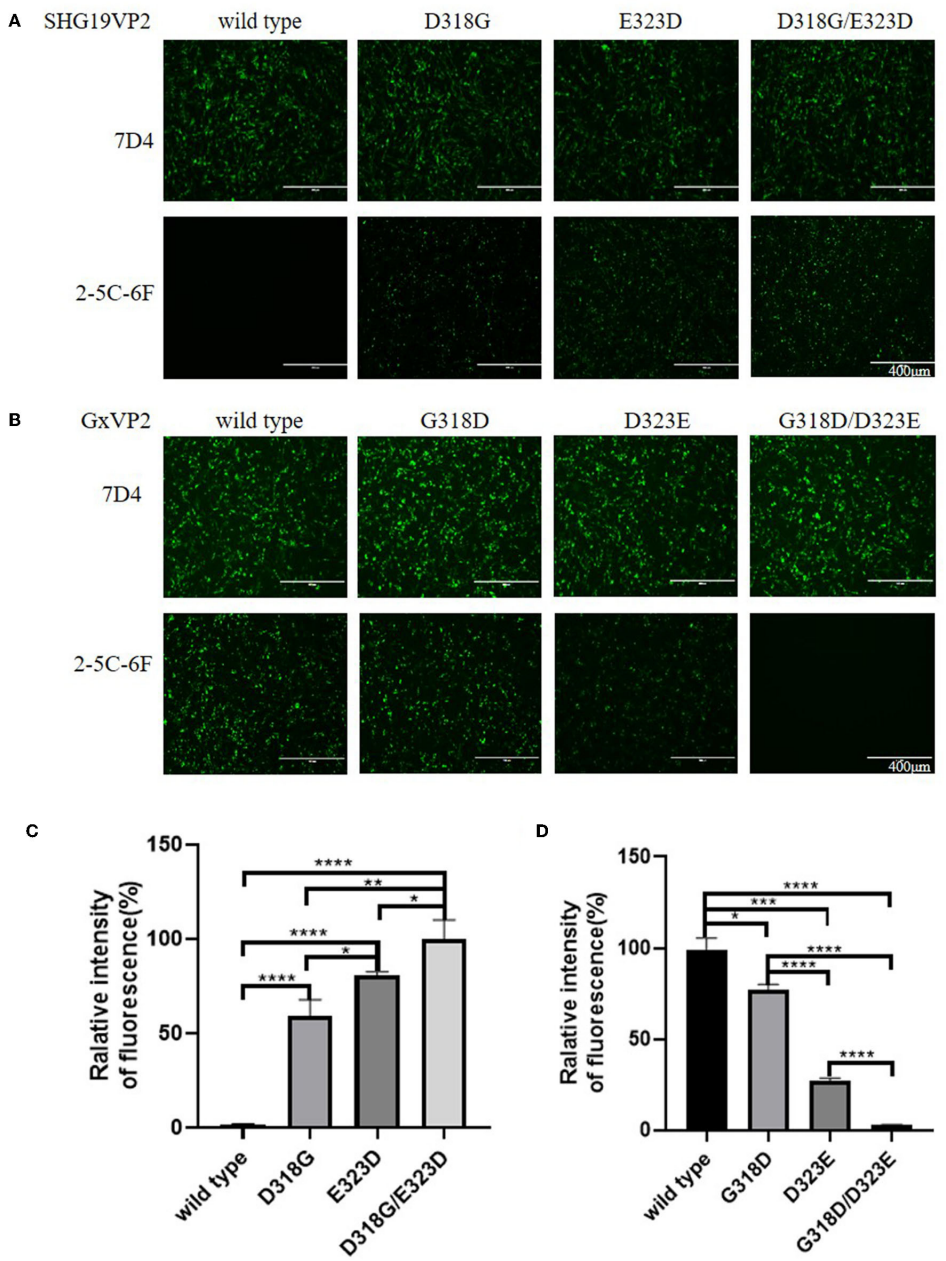
Antigen recognition detection of the wild and mutated types of VP2 in DF1 cells directed by MAbs 7D4 and 2-5C-6F. **(A)** VP2 of the SHG19 strain (SHG19VP2) and its mutated types (SHG19VP2-D318G, SHG19VP2-E323D, and SHG19VP2-D318G/E323D). **(B)** VP2 of the Gx strain (GxVP2) and its mutated types (GxVP2-G318D, GxVP2-D323E, and GxVP2- G318D/ D323E). **(C)** Quantitative analysis of fluorescence of **(A)**. **(D)** Quantitative analysis of fluorescence of **(B)**. The mean fluorescence intensity and standard deviations (error bars) from three independent samples are shown. * represents *P* < 0.05, ** represents *P* < 0.01, *** represents *P* < 0.001, **** represents *P* < 0.0001.

To determine the influence of residue mutations of 318 and 323 on the recognition of the MAb 2-5C-6F to viral VP2, three recombinant eukaryotic expression plasmids were transfected into DF1 cells to express the wild type of SHG19 VP2, its single-mutated type (SHG19VP2-D318G or SHG19VP2-E323D), and its double-mutated type (SHG19VP2-D318G/E323D). At 48 h post-transfection, the recognition of the MAb to viral VP2 was detected with IFA. The IFA results directed by MAb 7D4 confirmed the expression of all four VP2 mentioned before. The IFA results showed that MAbs 2-5C-6F did not recognize SHG19 VP2 but could recognize its single-mutated type (SHG19VP2-D318G or SHG19VP2-E323D) and its double-mutated type (SHG19VP2-D318G/E323D) (Figure 4A). The relative fluorescence intensity results confirmed this (Figure 4C). Conversely, MAbs 2-5C-6F could recognize Gx VP2 and its single-mutated type (GxVP2-G318D or GxVP2-D323E) but could not well recognize its double-mutated type (GxVP2-G318D/D323E) (Figures 4B,D). The data from both directions identified that residues mutations of 318 and 323 of VP2 was involved in the recognition of the MAb 2-5C-6F to viral VP2.

**Figure 5:**
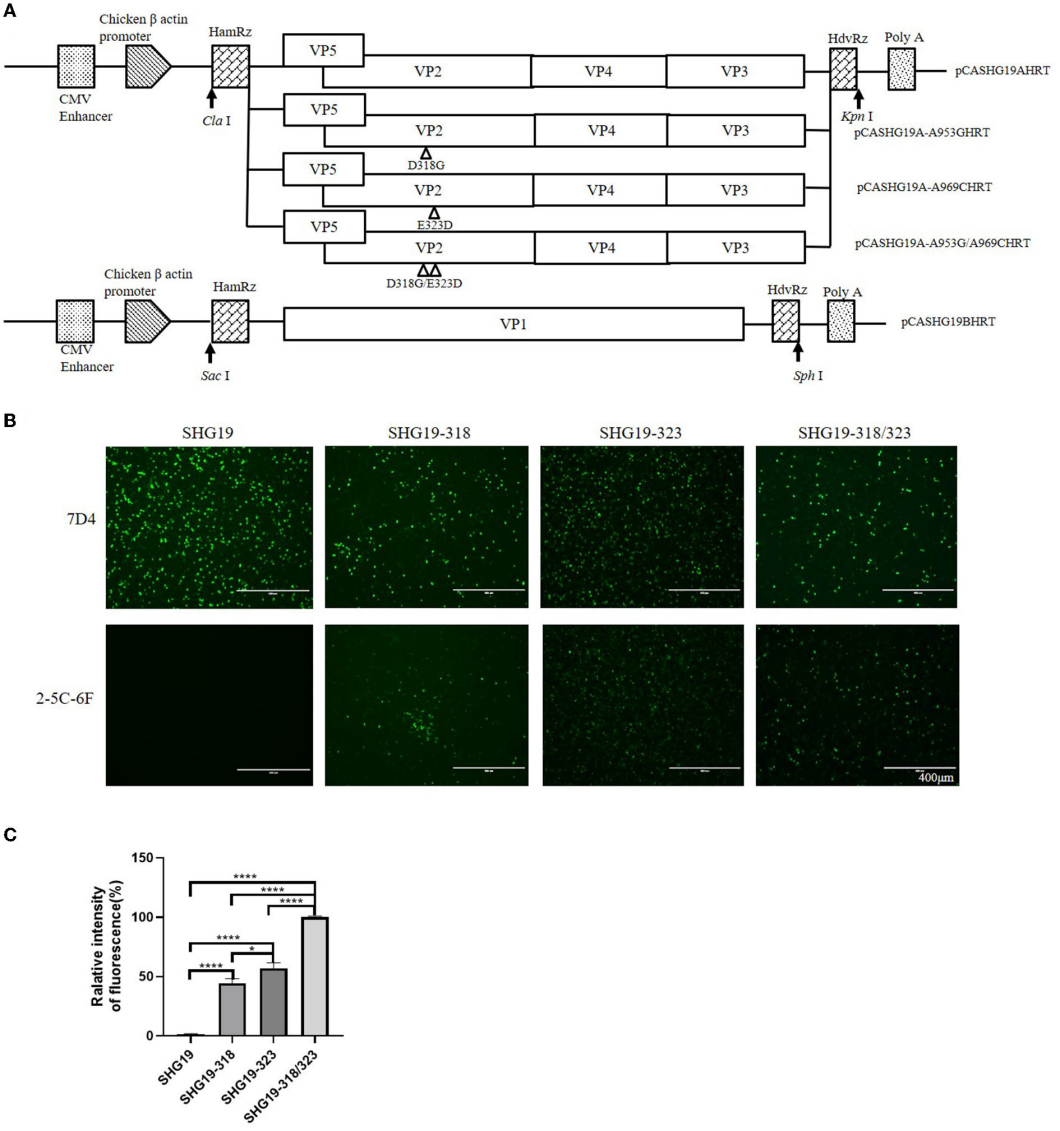
Antigen recognition detection of the epizootic varIBDV SHG19 and its mutants in DT40 cells directed by MAbs 7D4 and 2-5C-6F. **(A)** Schematic diagrams of the infectious clones containing segment A and segment B of SHG19. In plasmid pCASHG19A-A953GHRT, pCASHG19A-A969CHRT, and pCSHG19A-A953G/A969CHRT, the nucleotide substitutions A953G, A969C, and A953G/A969C resulted in the amino acid substitutions D318G, E323D, and D318G/E323D of the VP2 protein of SHG19, respectively. The genomic cDNA sequences are preceded by a cytomegalovirus enhancer and a chicken β-actin promoter and are flanked by the cDNAs of hammerhead ribozyme (HamRz) and hepatitis delta ribozyme (HdvRz). The restriction enzyme sites used to construct recombinant vectors are also shown. **(B)** IFA of SHG19 and its mutants (SHG19-318, SHG19-323, and SHG19-318/323) on DT40 cells directed by MAbs 7D4 and 2-5C-6F at 24 h post-infection. **(C)** Quantitative analysis of fluorescence. The mean fluorescence intensity and standard deviations (error bars) from three independent samples are shown. * represents *P* < 0.05, **** represents *P* < 0.0001.

To further verify the involvement of residues 318 and 323 of VP2 in the recognition of the MAb 2-5C-6F to the virus, the mutated IBDVs SHG19, SHG19-318, SHG19-323, and SHG19-318/323 were successfully rescued (Figure 5A), which was confirmed by RT-PCR, sequencing, and IFA (data not shown). The mutated viruses SHG19-318, SHG19-323, and SHG19-318/323 showed similar replication properties as the parental IBDV SHG19 (Supplementary Figure 2). In the following experiment of MAb recognition, at 24 h post-infection in DT40 cells, the recognition of MAb to viral VP2 was detected with IFA. The IFA results directed by MAb 7D4 confirmed the infection of all four IBDV mentioned before. The IFA results directed by the MAb 2-5C-6F showed that the MAb 2-5C-6F did not well recognize SHG19 but could recognize its single-mutated strain (SHG19-318 or SHG19-323) and its double-mutated strain (SHG19-318/323) (Figures 5B,C). These data from both aspects of protein and virus discovered that residues mutations of 318 and 323 of VP2 influenced the recognition of IBDV by the MAb.

### Residue mutations of 318 and 323 of VP2 influence the neutralization of IBDV by MAb

**Figure 6:**
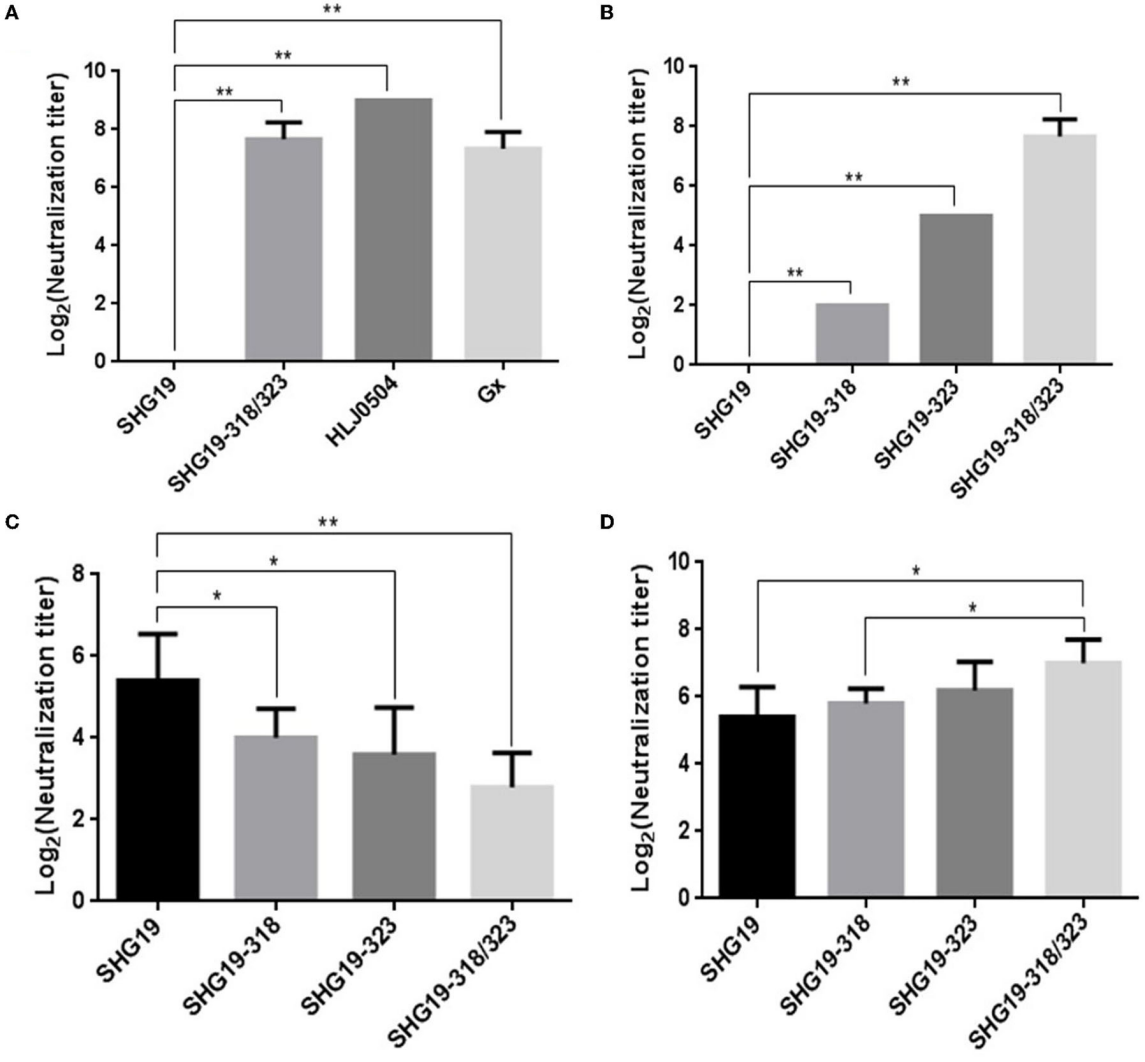
Cross-neutralizing assay. **(A)** Cross-neutralizing assay directed by the MAb 2-5C-6F for the epizootic varIBDV (SHG19 strain), vvIBDV (HLJ0504 strain and Gx strain), and double-mutated virus (SHG19-318/323). **(B)** Cross-neutralizing assay directed by the MAb 2-5C-6F for the SHG19 strain and its mutants (SHG19-318, SHG19-323, and SHG19-318/323). **(C)** Cross-neutralizing assay directed by SHG19 antiserum for the SHG19 strain and its mutants. **(D)** Cross-neutralizing assay directed by SHG19-318/323 antiserum for the SHG19 strain and its mutants. The mean titers and standard deviations (error bars) from three **(A,B)** or five **(C,D)** independent samples are shown. * and ** represent *P* < 0.05 and *P* < 0.01.

To further study whether residue mutations of 318 and 323 of VP2 influence the neutralization of IBDV by antiserum, cross-neutralizing assays on DT40 cells were performed. First, the neutralizing MAb 2-5C-6F against the vvIBDV was used. The neutralizing results showed that the MAb 2-5C-6F could neutralize the vvIBDV Gx strain and HLJ0504 strain, but could not neutralize the epizootic varIBDV SHG19. However, the MAb 2-5C-6F could neutralize the double-mutated SHG19 (SHG19-318/323) with a neutralization titer of 7.33 ± 0.58 log_2_ (Figure 6A). Furthermore, the neutralization ability of the MAb 2-5C-6F to single-mutated SHG19 (SHG19-318 or SHG19-323) was detected. the MAb 2-5C-6F could neutralize SHG19-318 and SHG19-323, with average neutralization titers of 2 ± 0 log_2_ and 5 ± 0 log_2_, which were 5.33 log_2_ and 2.33 log_2_ lower than those of SHG19-318/323, respectively (Figure 6B). These results showed that residue mutations of D318G/E323D were deeply involved in the neutralization circumvention of the epizootic varIBDV SHG19 from the MAb 2-5C-6F. In terms of affecting the ability of the MAb 2-5C-6F to neutralize the epizootic varIBDV SHG19, the double-mutated strain was more efficient than the single-mutated strain, and the residue 323 of VP2 played more important roles (Figure 6B).

### Residue mutations of 318 and 323 of VP2 influence the neutralization of IBDV by antiserum

The MAb targets only one antigen epitope, while antiserum contains antibodies against multiple antigenic epitopes. Although residues mutations of 318 and 323 of VP2 can affect the neutralization ability of its monoclonal antibody to the virus, is it enough to interfere with the neutralization ability of antiserum to virus? So the neutralization of the IBDV by antiserum was further identified. Compared with homologous neutralization to SHG19 by SHG19 antiserum (5.40 ± 1.14 log_2_), the neutralization titer to the single-mutated viruses SHG19-318 (4.00 ± 0.71 log_2_) and SHG19-323 (3.60 ± 1.14 log_2_) was obviously reduced by 1.40 log_2_ and 1.80 log_2_, respectively. For double-mutated virus (SHG19-318/323, 2.80 ± 0.84 log_2_), the neutralization titer of SHG19 antiserum was reduced more by 2.60 log_2_ (Figure 6C). Moreover, the SHG19-318/323 antiserum was used to detect the neutralization ability to different viruses. Compared with the homologous neutralization to SHG19-318/323 (7.00 ± 0.71 log_2_) by SHG19-318/323 antiserum, the neutralization titer to SHG19-323 (6.20 ± 0.84 log_2_), SHG19-318 (5.80 ± 0.45 log_2_), and the wild type of SHG19 (5.40 ± 0.89 log_2_) was reduced by 0.8 log_2_, 1.20 log_2_, and 1.60 log_2_, respectively (Figure 6D). The evidence from various angles identified that residues 318 and 323 were important factors affecting the cross-neutralization between the epizootic varIBDV and vvIBDV.

## Discussion

Since the late 1980s, the vvIBDV has spread globally and become one of the greatest threats to the healthy development of the poultry industry. With the use of vaccines and the improvement of feeding management, the vvIBDV is gradually being controlled, especially in large-scale intensive farms. However, since 2017, the atypical epizootic IBD has gradually become severe, mainly in poultry breeding areas of China (Fan et al., 2019; Xu A. et al., 2019; Jiang et al., 2021; Wang Y. et al., 2021), South Korea (Thai et al., 2021), Japan (Myint et al., 2021), and Malaysia (Aliyu et al., 2021). Although not fatal to chickens, atypical IBD causes severe atrophy of the central immune organs of the infected chickens, weakens host immunity, interferes with the immune response to other vaccines, causes complications and secondary infections by other pathogens, and reduces production performance including weight (Fan et al., 2019, 2020c; Xu A. et al., 2019; Li et al., 2020). The varIBDV appeared in the United States as early as the late 1980s and mainly circulated in North America. It is one of the important diseases severely threatening in the poultry industry in the United States (Ojkic et al., 2007; Stoute et al., 2019) and Canada (Amini et al., 2015). However, the newly epizootic varIBDV of the genotype A2bB1 in East Asia is genetically different from the early varIBDV circulating in North America (Fan et al., 2019; Wang Y. et al., 2021). In addition to the vvIBDV, the newly epizootic varIBDV has become another important threat to the healthy development of the poultry industry at least in China (Jiang et al., 2021; Wang Y. et al., 2021).

In China, to prevent and control the vvIBDV, almost all chicken flocks are immunized with vaccines including live vaccine, subunit vaccine, immune complex vaccine, and combined vaccine. Interestingly, what are the reasons for the wild spread of the epizootic VarIBDV of the genotype A2bB1 in immunized chicken flocks? Sequence analysis showed that the epizootic VarIBDV had the same characteristic amino acids as the reference strain of the early VarIBDV (variant E), including 213N, 222T, 242V, 249K, 253Q, 279N, 284A, 286I, 294L, 318D, 323E, and 330S, which is an indication of possible antigenic changes compared to the vvIBDV. The cross-neutralization test in this study proved the speculation that the neutralizing ability of the vvIBDV antiserum to the epizootic varIBDV decreased by 7 log_2_, and the average neutralizing titer of the epizootic varIBDV antiserum to vvIBDV decreased by 3.2 log_2_. In our next plan, the immune protection of the available vvIBDV vaccine against the epizootic varIBDV will be continuously monitored. However, our current research shows that enough attention should be paid to the application of the cross-neutralization test to evaluate the immune protection spectrum of vaccine in advance. In addition, the MAb 2-5C-6F, which had good neutralizing activity against the vvIBDV, could not recognize the epizootic VarIBDV well. These results discovered that the epizootic varIBDV had different antigenicity from the vvIBDV.

What is the molecular basis of the antigenic difference between the epizootic varIBDV and vvIBDV? First, the epitope of the neutralizing MAb 2-5C-6F of the vvIBDV was identified to be located at aa 317-336 of the P_HI_ of the capsid protein VP2. The P_HI_ is an important domain for immunoreactivity (Lee et al., 2006; Letzel et al., 2007). Compared with the vvIBDV, the epizootic varIBDV had two amino acid differences in this epitope, G318D, and D323E. Subsequently, the IFA based on protein mutants of VP2 showed that the double-mutations of G318D/D323E made the VP2 of the vvIBDV no longer be well recognized by the MAb 2-5C-6F, while the D318G or/and E323D mutations could make the epizootic varIBDV acquire the ability to be recognized by the MAb 2-5C-6F. Furthermore, using mutated viruses, it was further confirmed that the mutations of D318G, E323D, or D318G/E323D could enable the unrecognized epizootic varIBDV to acquire the ability to be recognized by the MAb 2-5C-6F. These data indicated that aa 318 and aa 323 of VP2 were important molecular bases for the antigenic difference between the epizootic varIBDV and vvIBDV.

Are the antigenicity changes induced by aa 318 and aa 323 mutations enough to affect the antiserum viral neutralization ability? For the first time, our study showed that the MAb 2-5C-6F could neutralize the vvIBDV but had little neutralizing activity against the epizootic varIBDV. When residues 318G and 323D of the epizootic varIBDV were mutated to 318D and 323E of the vvIBDV, 2-5C-6F could neutralize the corresponding mutant viruses, and its ability to neutralize the double-mutated virus (SHG19-318/323) was stronger than that of the single-mutated virus (SHG19-318 or SHG19-323). It is well known that antiserum targets various antigen epitopes, while the MAb only targets one, so the mutations that can escape the MAb might not be enough to escape antiserum. Furthermore, the viral neutralizing ability changes of the SHG19 antiserum were evaluated. Results showed that compared to the parental strain SHG19, the ability to neutralize single-mutated viruses (SHG19-318 or SHG19-323) was significantly downregulated, and the ability to neutralize the double-mutated virus (SHG19-318/323) was further downregulated by 2.60 log_2_. On the contrary, compared to SHG19-318/323, the neutralizing titer of SHG19-318/323 antiserum to SHG19 was also reduced by 1.60 log_2_. About the effect on MAb or antiserum viral recognition and neutralization ability, residue 323 was stronger than residue 318, and double-mutation was greater than single-mutation. According to the predicted VP2 structure, residues 318 and 323 are located on the outermost region of viral capsid protein VP2, and the mutations G318D and D323E might alter the structure and the associated electrostatic potential.

Both aspects of data identified that the mutations of residues 318 and 323 of VP2 significantly affected the recognition and neutralization of IBDV by antiserum, which might be deeply involved in the immune circumvention of the epizootic varIBDV in the vaccinated flock. To be mentioned, the newly epizootic varIBDV exhibits the same amino acids as the early varIBDV variant E strain at positions 318 and 323 of VP2, which is a case for the selection of converging antigenically significant mutations. It was reported that aa 318-324 of VP2 were critical for vvIBDV typical and atypical antigenicity (Eterradossi et al., 1998). Letzel et al. (2007) found that residues 318 and 323 of VP2 were critical for the reactivity of MAb 10 (Letzel et al., 2007). It has been also reported that residue 323 might influence the binding of more than one MAb to the IBDV directly or indirectly (Vakharia et al., 1994; Letzel et al., 2007). In this study, it was further proved that residues 318 and 323 of VP2 could affect the neutralizing activity of antiserum, which was involved in the immune circumvention of the epizootic varIBDV in the vaccinated flock. Our research not only further proved the function of these key mutation hotspots but also clarified the important molecular mechanism of the epizootic varIBDV prevalence. In addition, these key mutation hotspots including residues 318 and 323 of VP2 could be mentioned as a good indication of possible antigenic changes.

Modern molecular biology techniques such as reverse genetics make it possible to edit vaccine strains manually (Yu et al., 2016; Fan et al., 2020b). To develop new vaccines that match the antigenicity of varIBDV, the key residues involved in immune circumvention should be taken into account. A recent research indicated that the emerging varIBDV and the persistently circulating vvIBDV are two important threats (Jiang et al., 2021). So the currently used vvIBDV vaccine needs to continue to be used scientifically. It is a more meaningful to develop broad-spectrum vaccines that can prevent both varIBDV and vvIBDV.

## Conclusion

This study revealed a significant difference in antigenicity between the epizootic varIBDV and vvIBDV and further proved that residues 318 and 323 of the VP2 protein P_HI_ interfered with antiserum viral neutralization, which provided insights into one important reason for the prevalence of the epizootic varIBDV in immunized chickens. This study is significant for the comprehensive prevention and control of the emerging varIBDV.

## Data availability statement

The raw data supporting the conclusions of this article will be made available by the authors, without undue reservation.

## Ethics statement

The animal study was reviewed and approved by Ethics and Animal Welfare Committee of HVRI.

## Author contributions

XQ, LF, and YW contributed conception and design of the study. LF and YW performed the experiments and wrote the manuscript. NJ and XN performed the relevant experiments. WZ, MH, and KB analyzed the data. AL and SW contributed to figures. YG, LG, KL, HC, QP, CL, YZ, and XW advised on experimental design and data interpretation. XQ supervised the study, interpreted the data, wrote the manuscript, and acquired the research funds. All authors contributed to the article and approved the submitted version.

## Funding

This study was supported by the National Natural Science Foundation of China (Grant Nos. 32072852 and 32102649), the Heilongjiang Provincial Natural Science Foundation of China (Grant Nos. ZD2020C006 and TD2019C003), the Key Research and Development Program of Heilongjiang Province (Grant No. GA21B004), and China Agriculture Research System (CARS-41-G15).

## Conflict of interest

The authors declare that the research was conducted in the absence of any commercial or financial relationships that could be construed as a potential conflict of interest.

## Publisher's note

All claims expressed in this article are solely those of the authors and do not necessarily represent those of their affiliated organizations, or those of the publisher, the editors and the reviewers. Any product that may be evaluated in this article, or claim that may be made by its manufacturer, is not guaranteed or endorsed by the publisher.

## Supplementary material

Supplementary Figure 1 Characteristic amino acid substitutions in VP2 among varIBDV strains isolated from broiler, layer, and local breed chickens. Asterisks indicate residues identical to the sequence of varIBDV strain SHG19. Residues 318 and 323 were highlighted.

Supplementary Figure 2 The replication of the mutated IBDV in DT40 cells, and the viral genome copies were detected at 24, 36, 48, and 60 hours post-infection by RT-qPCR. The mean fluorescence intensity and standard deviations (error bars) from three independent samples are shown.
