# Supplementary material for: Residues 318 and 323 in capsid protein are involved in immune circumvention of the atypical epizootic infection of infectious bursal disease virus
Source: Front Microbiol. 2022 Jul 29;13:909252. doi: 10.3389/fmicb.2022.909252 (PMC9372508; doi:10.3389/fmicb.2022.909252)
Supplement: Supplementary file 1 [file Table_1.docx]

Supplement Table 1 Primers used in antigen epitope identification.

| Primer | Sequence | Orientation |
| --- | --- | --- |
| VP2-1F | GGTT**GGATCC**ACGAACCTGCAAGATCAAAC | Sense |
| VP2-1R | TTAA**CTCGAG**GACGGTTACCCCTTCCCCTAC | Antisense |
| VP2-2F | GGTT**GGATCC**TACAATGGGTTGATGTCTGC | Sense |
| VP2-2R | TTAA**CTCGAG**GATTGGCTGGGTTATCTCGC | Antisense |
| VP2-3F | TTTAAA**GGATCC**GGGCTAACGGCCGGCACTGAC | Sense |
| VP2-3R | TTTTGG**CTCGAG**TGCTCCTGCAATCTTCAGGGGAG | Antisense |
| VP2-4F | GGTT**GGATCC**TACAATGGGTTGATGTCTGC | Sense |
| VP2-4R | TTAA**CTCGAG**GTAGACTCTGGGCCTGTCAC | Antisense |
| VP2-5F | GGTT**GGATCC**GACCCAAAAATGGTAGCAAC | Sense |
| VP2-5R | TTAA**CTCGAG**AAGGCCTTGGACGCTTGTTTG | Antisense |
| VP2-6F | TTTTAA**GGATCC**CTCAGCATCGGGGGAGAAC | Sense |
| VP2-6R | TTAAC**TCGAGG**ATTGGCTGGGTTATCTCGC | Antisense |
| VP2-7F | TTTTAAA**GGATCC**GGGCTAACGGCCGGCACTG | Sense |
| VP2-7R | TTTAAA**CTCGAG**GAGGGCCCCTGGATAGTTGC | Antisense |
| VP2-8F | TTTAAA**GGATCC**AGTGGGAGCCTAGCAGTGAC | Sense |
| VP2-8R | TTAA**CTCGAG**CATGGCTCCTGGGTCAAATC | Antisense |
| VP2-9F | TTAA**GGATCC**GCAAAGAACCTGGTCACAG | Sense |
| VP2-9R | TTAA**CTCGAG**TGCTCCTGCAATCTTCAGGG | Antisense |
| VP2-10F | GGTT**GGATCC**TACAATGGGTTGATGTCTGC | Sense |
| VP2-10R | TTAA**CTCGAG**GGGTAAGCTGAGGACGGTTAC | Antisense |
| VP2-11F | TTTT**GGATCC**CTAGTAGGGGAAGGGGTAAC | Sense |
| VP2-11R | TTTT**CTCGAG**CCCTATAGCGGGAATGGGGTC | Antisense |
| VP2-12F | TTTT**GGATCC**TATGTGAGACTCGGTGACCC | Sense |
| VP2-12R | TTTT**CTCGAG**GTAGACTCTGGGCCTGTCACTG | Antisense |
| VP2-13F | TTTTAAA**GGATCC**GGGCTAACGGCCGGCACTGAC | Sense |
| VP2-13R | TTGG**CTCGAG**CAGTTTGATGGATGTGATTGG | Antisense |
| VP2-14F | TTAA**GGATCC**AGCGAGATAACCCAGCC | Sense |
| VP2-14R | TTAA**CTCGAG**TGACCATGACATCTGGTC | Antisense |
| VP2-15F | TTTTAAAA**GGATCC**AGTGGTGGTCAGGCGGGGGAC | Sense |
| VP2-15R | TTTAAA**CTCGAG**GAGGGCCCCTGGATAGTTGC | Antisense |
| VP2-16F | **GATCC**AGTGGTGGTCAGGCGGGGGACCAGATGTCATGGTCAGCAAGTGGGAGCCTAGCAGTGACG**C** | Sense |
| VP2-16R | **TCGAG**CGTCACTGCTAGGCTCCCACTTGCTGACCATGACATCTGGTCCCCCGCCTGACCACCACT**G** | Antisense |
| VP2-17F | **GATCC**TGGTCAGCAAGTGGGAGCCTAGCAGTGACGATCCACGGTGGCAACTATCCAGGGGCCCTC**C** | Sense |
| VP2-17R | **TCGAG**GAGGGCCCCTGGATAGTTGCCACCGTGGATCGTCACTGCTAGGCTCCCACTTGCTGACCA**G** | Antisense |
| VP2-18F | **GATCC**GGTGGTCAGGCGGGGGACCAGATGTCATGGTCAGCAAGTGGGAGCCTAGCAGTGACG**C** | Sense |
| VP2-18R | **TCGAG**CGTCACTGCTAGGCTCCCACTTGCTGACCATGACATCTGGTCCCCCGCCTGACCACC**G** | Antisense |
| VP2-19F | **GATCC**GGTCAGGCGGGGGACCAGATGTCATGGTCAGCAAGTGGGAGCCTAGCAGTGACG**C** | Sense |
| VP2-19R | **TCGAG**CGTCACTGCTAGGCTCCCACTTGCTGACCATGACATCTGGTCCCCCGCCTGACC**G** | Antisense |
| VP2-20F | **GATCC**AGTGGTGGTCAGGCGGGGGACCAGATGTCATGGTCAGCAAGTGGGAGCCTAGCAGTG**C** | Sense |
| VP2-20R | **TCGAG**CACTGCTAGGCTCCCACTTGCTGACCATGACATCTGGTCCCCCGCCTGACCACCACT**G** | Antisense |
| VP2-21F | **GATCC**CTCGGTGACCCCATTCCCGCTATAGGGCTCGACCCAAAAATGGTAGCAACA**C** | Sense |
| VP2-21R | **TCGAG**TGTTGCTACCATTTTTGGGTCGAGCCCTATAGCGGGAATGGGGTCACCGAGG**G** | Antisense |
| VP2-22F | **GATCC**TATGTGAGACTCGGTGACCCCATTCCCGCTATAGGGCTCGACCCAAAAATG**C** | Sense |
| VP2-22R | **TCGAG**CATTTTTGGGTCGAGCCCTATAGCGGGAATGGGGTCACCGAGTCTCACATA**G** | Antisense |
| VP2-23F | **GATCC**CCCATTCCCGCTATAGGGCTCGACCCAAAAATGGTAGCAACA**C** | Sense |
| VP2-23R | **TCGAG**TGTTGCTACCATTTTTGGGTCGAGCCCTATAGCGGGAATGGG**G** | Antisense |
| VP2-24F | **GATCC**TATGTGAGACTCGGTGACCCCATTCCCGCTATAGGGCTCGAC**C** | Sense |
| VP2-24R | **TCGAG**GTCGAGCCCTATAGCGGGAATGGGGTCACCGAGTCTCACATA**G** | Antisense |
| VP2-25F | **GATCC**CCCATTCCCGCTATAGGGCTCGACCCAAAAATGGTA**C** | Sense |
| VP2-25R | **TCGAG**TACCATTTTTGGGTCGAGCCCTATAGCGGGAATGGG**G** | Antisense |
| VP2-26F | **GATCC**CCCATTCCCGCTATAGGGCTCGACCCAAAAATG**C** | Sense |
| VP2-26R | **TCGAG**CATTTTTGGGTCGAGCCCTATAGCGGGAATGGG**G** | Antisense |
| VP2-27F | **GATCC**CCCATTCCCGCTATAGGGCTCGACCCAAAA**C** | Sense |
| VP2-27R | **TCGAG**TTTTGGGTCGAGCCCTATAGCGGGAATGGG**G** | Antisense |
| VP2-28F | **GATCC**CCCATTCCCGCTATAGGGCTCGACCCA**C** | Sense |
| VP2-28R | **TCGAG**TGGGTCGAGCCCTATAGCGGGAATGGG**G** | Antisense |
| VP2-29F | **GATCC**ATTCCCGCTATAGGGCTCGACCCAAAAATGGTAGCAACA**C** | Sense |
| VP2-29R | **TCGAG**TGTTGCTACCATTTTTGGGTCGAGCCCTATAGCGGGAAT**G** | Antisense |
| VP2-30F | **GATCC**CCCGCTATAGGGCTCGACCCAAAAATGGTAGCAACA**C** | Sense |
| VP2-30R | **TCGAG**TGTTGCTACCATTTTTGGGTCGAGCCCTATAGCGGG**G** | Antisense |

Note: Primers were designed according to the sequence of IBDV strain Gx (GenBank accession no. AY444873). The introduced restriction sites were highlighted with underlines.
