# Supplementary figures and images for: Residues 318 and 323 in capsid protein are involved in immune circumvention of the atypical epizootic infection of infectious bursal disease virus
Source: Front Microbiol. 2022 Jul 29;13:909252. doi: 10.3389/fmicb.2022.909252 (PMC9372508; doi:10.3389/fmicb.2022.909252)

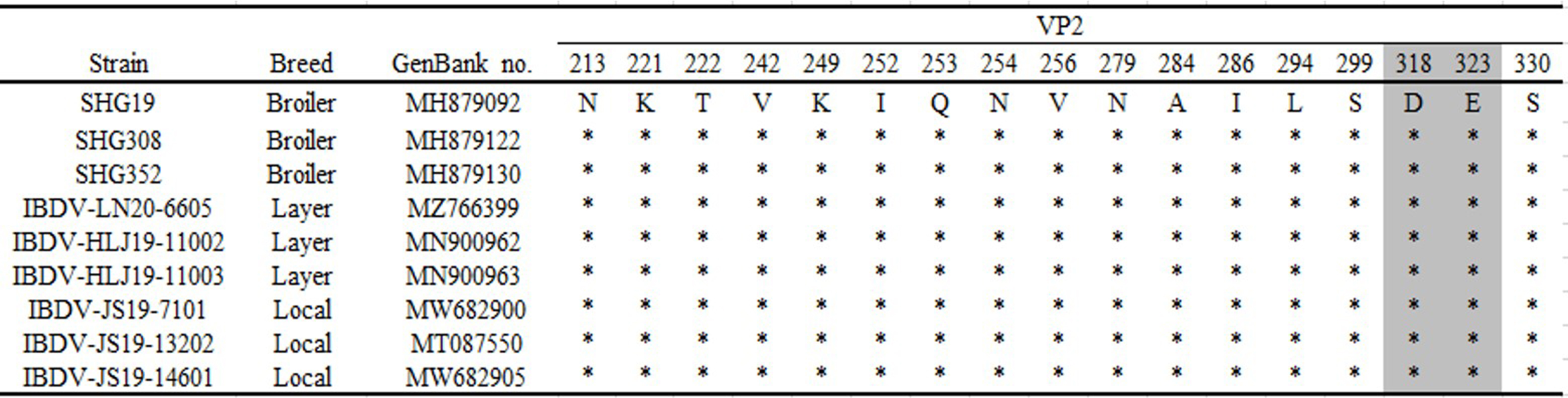

Supplement: Supplementary Figure 1 — Characteristic amino acid substitutions in VP2 among varIBDV strains isolated from broiler, layer, and local breed chickens. Asterisks indicate residues identical to the sequence of varIBDV strain SHG19. Residues 318 and 323 were highlighted. [file Image_1.jpeg]

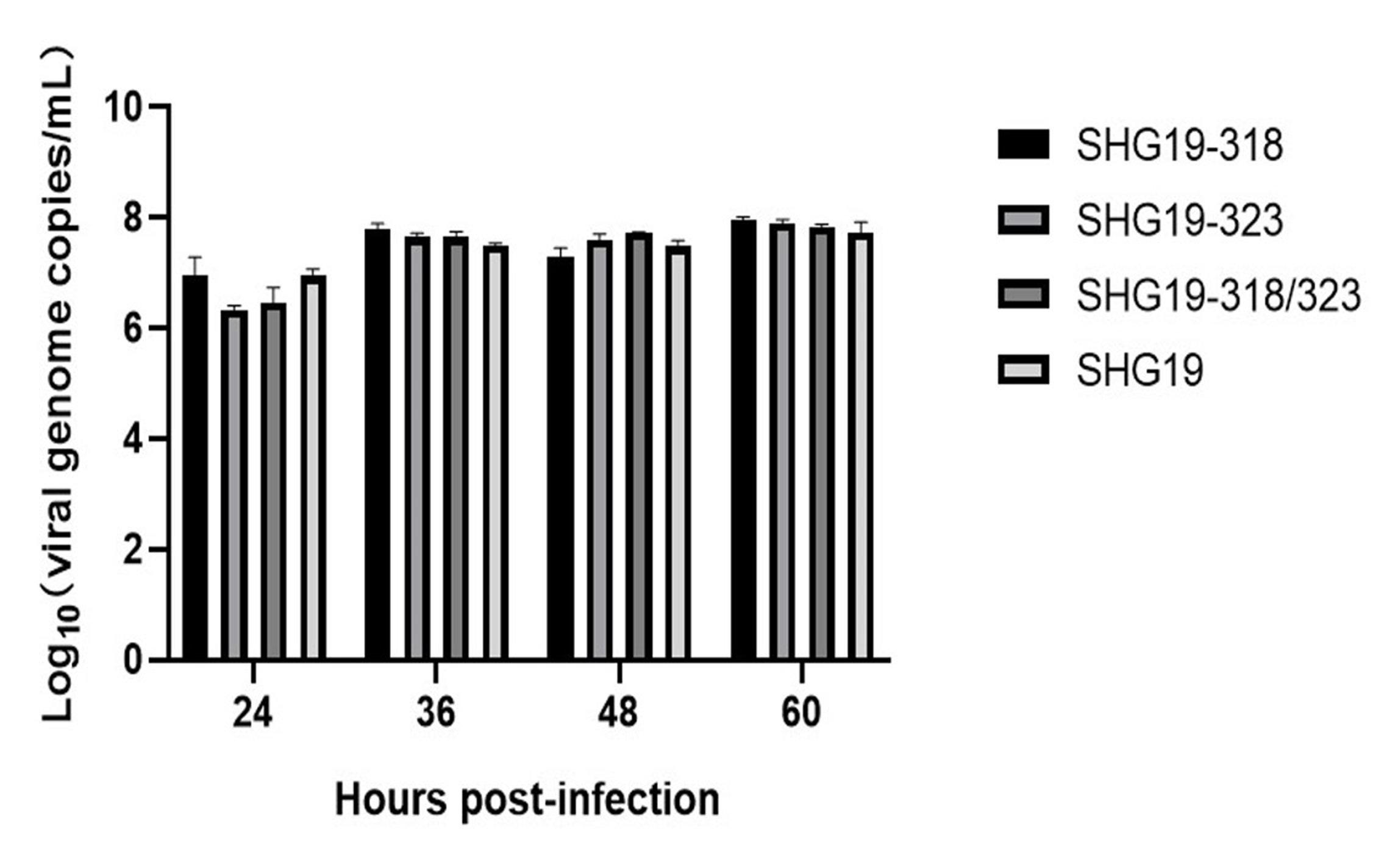

Supplement: Supplementary Figure 2 — The replication of the mutated IBDV in DT40 cells, and the viral genome copies were detected at 24, 36, 48, and 60 hours post-infection by RT-qPCR. The mean fluorescence intensity and standard deviations (error bars) from three independent samples are shown. [file Image_2.jpeg]
